# Screening, molecular identification, and evaluation the effects of indigenous Plant Growth-Promoting Rhizobacteria on growth indices and nutrient uptake of chamomile (*Matricaria chamomilla*) under saline conditions

**DOI:** 10.3389/fmicb.2025.1551310

**Published:** 2025-05-13

**Authors:** Seyed Hassan Tafaroji, Seyed Ali Abtahi, Mojtaba Jafarinia, Mehdi Ebadi

**Affiliations:** ^1^Department of Soil Science, Marv.C., Islamic Azad University, Marvdasht, Iran; ^2^Department of Biology, Marv.C., Islamic Azad University, Marvdasht, Iran; ^3^Department of Microbiology, Lar.C., Islamic Azad University, Larestan, Iran

**Keywords:** salinity, PGPR, *Matricaria chamomilla*, rhizosphere soil, growth indices, 16SrRNA

## Abstract

**Introduction:**

Salinity is a major issue affecting agricultural lands, leading to reduced crop productivity and soil degradation. One approach to mitigate the effects of salinity is utilizing PGPR. This study aimed to isolate and identify indigenous PGPR from rhizosphere soil and evaluate their effects on the growth indices of chamomile under saline conditions.

**Methods:**

Forty-five rhizosphere soil samples were collected from agricultural fields in Beyram, Iran. The PGPR were isolated and identified using standard phenotypic, biochemical, and molecular assays. Plant growth-promoting traits were applied for PGPR strain screening. The effects of selected PGPR strains on the growth indices and nutrient uptake of chamomile under saline conditions were evaluated in a greenhouse experiment.

**Results and discussion:**

A total of 181 bacterial isolates were identified from the 45 soil samples, belonging to eight genera and 13 species. Seven species, including *B. cereus, P. fluorescens, P. syringae, A. radiotolerans, P. phenanthrenivorans, P. alcaliphila*, and *L. macroides*, possessing all five growth-promoting characteristics, were selected for further experiments. PGPR treatments significantly improved chamomile's growth, biochemical parameters, and nutrient uptake under different salinity levels. The P1 treatment at 2 dS.m^−1^ salinity had the highest root (16.75 cm) and shoot length (32.91 cm), along with dry root (0.089 g) and shoot weight (1.67 g). Biochemical improvements included higher chlorophyll and essential oil content with P2 at 2 dS.m^−1^. Increased salinity decreased overall plant growth and nutrient uptake. The indigenous PGPR strains showed promising potential to enhance chamomile growth and nutrient status under salt stress, offering a sustainable strategy for improving crop productivity in saline-affected regions.

## Introduction

Salinity is a major issue affecting agricultural lands, particularly in arid and semi-arid regions, leading to reduced crop productivity and soil degradation. High salinity levels in the soil disrupt plant water uptake by creating an osmotic imbalance, cause ion toxicity due to excessive sodium (Na^+^) and chloride (Cl^−^) accumulation, and lead to nutrient imbalances by interfering with the uptake of essential elements such as potassium (K^+^), calcium (Ca^2^^+^), and magnesium (Mg^2^^+^), ultimately inhibiting plant growth and reducing crop yield (Etikala et al., 2023). To mitigate the detrimental effects of salinity, several strategies can be employed, including the use of salt-tolerant crop varieties, the application of soil amendments such as gypsum and organic matter, and the implementation of improved irrigation practices, such as drip irrigation and leaching techniques, to manage salt accumulation in the root zone (Mishra et al., 2023).

One promising and sustainable approach to enhancing plant resilience under saline conditions is the utilization of Plant Growth-Promoting Rhizobacteria (PGPR). These bacteria enhance plant tolerance to salinity through various mechanisms, such as producing phytohormones [e.g., indole-3-acetic acid (IAA), gibberellins, and cytokinins] that promote root elongation and lateral root formation, solubilizing essential nutrients like phosphorus (P) and iron (Fe), and inducing systemic resistance to abiotic stress by modulating antioxidant enzyme activities and osmolyte accumulation (Hasan et al., 2024).

PGPR are beneficial soil bacteria that enhance plant growth and stress tolerance through multiple mechanisms, including nitrogen fixation, phosphate solubilization, phytohormone production, and biocontrol of soilborne pathogens. The application of PGPR in agriculture has gained considerable attention as a sustainable strategy to improve crop productivity and mitigate stress effects in challenging environments (Hasan et al., 2024). These bacteria have demonstrated the ability to enhance plant growth by improving nutrient uptake efficiency, producing growth-stimulating compounds such as exopolysaccharides (EPS) and 1-aminocyclopropane-1-carboxylate (ACC) deaminase, and inducing systemic resistance against fungal and bacterial pathogens.

Indigenous PGPR, naturally occurring in local soils, offer distinct advantages over introduced strains. They are better adapted to the native soil physicochemical conditions, salinity levels, and environmental stresses, making them more effective in promoting plant growth in their native ecosystems. The utilization of indigenous PGPR strains can provide a sustainable and eco-friendly approach to improving plant growth, particularly in stress-prone environments, by enhancing soil microbial diversity, facilitating soil structure improvement, and contributing to long-term soil fertility and stability (Agbodjato and Babalola, 2024).

In Iran, with its diverse climatic zones and agricultural practices, the potential of PGPR is highly significant. The country's soil diversity, shaped by its varied topography and climate, supports a rich microbial ecosystem (Motallebirad et al., 2023). PGPR isolated from Iranian soils have demonstrated considerable potential in promoting plant growth and enhancing stress tolerance. For instance, in Iran's arid and semi-arid regions, characterized by low rainfall and high salinity, PGPR have exhibited beneficial traits such as salt tolerance, drought resistance, and phosphate solubilization. These characteristics are crucial for improving crop productivity under harsh environmental conditions.

The isolation of indigenous PGPR strains is particularly valuable due to their adaptation to local soil and environmental conditions, which enhances their effectiveness compared to introduced strains. Additionally, studying PGPR from Iranian soils contributes to a broader understanding of plant-microbe interactions under various stress conditions, providing valuable insights for developing resilient crop management strategies (Gasemi et al., 2023). Therefore, by focusing on indigenous strains, it is possible to harness the natural benefits of these bacteria to address challenges such as salinity, drought, and other environmental stressors, thereby promoting more resilient and sustainable agricultural practices.

Chamomile (*Matricaria chamomilla*) is renowned for its medicinal properties and has been utilized for centuries in traditional medicine due to its anti-inflammatory, antioxidant, and soothing effects. As an economically important plant, chamomile is cultivated globally for its essential oils and extracts, which are widely used in pharmaceuticals, cosmetics, and herbal teas. However, chamomile cultivation faces significant challenges, particularly under saline conditions, which are increasingly prevalent due to soil salinization resulting from irrigation practices and climate change (Akram et al., 2024).

Salinity imposes multiple physiological and biochemical stresses on plants, including osmotic stress, ion toxicity, and nutrient imbalances. In chamomile, these adverse effects can lead to stunted growth, reduced biomass production, and a decline in essential oil content, ultimately affecting its medicinal and commercial value (Hendawy et al., 2019). Given these challenges, exploring the role of PGPR in mitigating salinity stress in chamomile cultivation is of great importance, as these beneficial microbes may offer an effective, sustainable solution for improving plant health and productivity under saline conditions.

Due to the high salinity levels in over 80% of Iran's land, where vast desert areas dominate the landscape, soil salinization poses a significant challenge to sustainable agriculture. The accumulation of salts in the soil disrupts plant growth, reduces crop productivity, and limits the cultivation of economically valuable plants, such as chamomile. Therefore, effective strategies for salt neutralization and soil quality improvement are essential to enhance agricultural sustainability in these regions. This study introduces an innovative approach by isolating and identifying indigenous PGPR strains from diverse environmental sources and evaluating their potential in mitigating the adverse effects of salinity on chamomile. Unlike previous studies that primarily focused on non-native bacterial strains or model plants, our research emphasizes the use of naturally adapted indigenous PGPR, which are more suited to the local soil and climatic conditions. Additionally, the mechanisms by which these bacteria enhance plant growth, including nutrient solubilization, phytohormone production, and stress tolerance induction, are investigated in detail. By examining the effects of PGPR on chamomile growth parameters under saline conditions, this study provides novel insights into their potential application for improving soil fertility, enhancing plant resilience, and promoting sustainable agricultural practices in salt-affected regions.

## Materials and methods

### Sampling and isolation of PGPR

To ensure the reliability and representativeness of the collected samples, a total of 45 rhizosphere soil samples were obtained from different agricultural fields in Beyram, Iran (27°24′58.3″ N, 53°32′43.6″ E, 506.2 meters above sea level). The soil type in the sampling sites was determined as sandy loam, based on soil texture analysis. The sampled fields had a history of conventional agriculture, with crop rotations involving wheat (*Triticum aestivum*), barley (*Hordeum vulgare*), alfalfa (*Medicago sativa*), and chamomile (*Matricaria chamomilla*). Minimal pesticide and fertilizer applications were reported, which likely influenced the native microbial communities. Understanding these factors was essential for interpreting the microbial diversity and plant growth-promoting potential of the isolated PGPR strains.

The sampling was conducted following a standard microbiological and agronomic method (Rehan et al., 2023) during the growing season (May–September 2020). At each sampling site, three independent replicates were collected from the rhizosphere zone at a depth of 0–20 cm, which is considered optimal for assessing root-associated microbial communities. Each replicate consisted of 5–10 g of soil tightly adhering to the roots of healthy plants, which were carefully excavated to minimize disturbance. Only healthy plants free from visible pathogens or external contaminants were selected to ensure an accurate assessment of microbial populations.

The sampling process involved the careful excavation of plants to expose the roots while minimizing damage. Loose soil was gently shaken off, and only the soil tightly adhering to the roots (rhizosphere soil) was retained. A portion of the root along with the adhering soil was cut with sterilized scissors. These root-soil samples were placed into sterile polyethylene bags to prevent contamination and minimize exposure to environmental factors. The samples were promptly transported to the microbiology laboratory at Marvdasht Islamic Azad University in an icebox and stored at 4°C until further processing.

The physicochemical properties of the soil, including pH, electrical conductivity (EC, dS/m), total nitrogen (N, %), available phosphorus (P, mg/kg), salinity (g/kg), and calcium carbonate (CaCO3, %), were measured following standard protocols (Fazelikia et al., 2023). The detailed properties of the collected soil samples are presented in Table 1.

**Table 1:** Physicochemichal properties of soil samples.

**Sample no**.	**Vegetation type**	**K (ppm)**	**P (ppm)**	**Total nitrogen (mg/kg)**	**Electrical conductivity (MS)**	**pH**	**CaCO_3_ (%)**	**Salinity (dS/m)**
1	Wheat	252	0.5	35	120.9	8.1	46.7	2.71
2	Alfalfa	234	11	43	118	8.4	53.4	3.75
3	Lettuce	196	8	75	131.6	7.9	49.4	2.81
4	Mallow	268	0.7	25	124	8.4	47.2	5.22
5	Wheat	25	0.1	24	118.3	8.0	51.1	6.97
6	Atmosphere	214	32	65	145	7.9	60.4	5.77
7	Spinach	185	12	45	110	8.5	55.1	4.21
8	Canola	222	0.8	78	98.3	8.3	43.6	3.73
9	Barley	236	6	26	146	8.4	48.4	7.14
10	Wheat	281	0.9	39	125	8.1	53.8	5.11
11	Canola	263	1.2	18	164	8.0	61.1	6.52
12	Beet	214	1.6	24	184	8.3	45.0	5.37
13	Barley	198	5	63	112	8.2	57.9	6.85
14	Mallow	225	2.3	18	95	8.3	43.8	3.98
15	Wheat	212	1.6	89	116	8.0	42.1	4.84
16	Canola	248	25	74	168	8.1	41.2	5.22
17	Wheat	226	18	63	133	7.7	40.5	6.05
18	Canola	289	1.8	42	101.3	7.9	49.2	4.16
19	Wheat	251	2.2	33	116	8.0	55.7	5.61
20	Spinach	255	0.9	28	95.5	8.5	51.3	8.12
21	Barley	252	0.5	35	120.9	8.4	58.8	6.26
22	Barley	234	11	43	118	8.1	51.6	7.10
23	Wheat	196	8	75	131.6	8.0	46.3	5.72
24	Alfalfa	268	0.7	25	124	8.2	46.4	3.17
25	Alfalfa	25	0.1	24	118.3	8.3	39.7	2.75
26	Barley	214	32	65	145	8.1	47.2	3.70
27	Wheat	185	12	45	110	8.3	41.3	3.12
28	Alfalfa	222	0.8	78	98.3	8.6	48.0	3.62
29	Canola	236	6	26	146	7.7	38.9	4.78
30	Alfalfa	281	0.9	39	125	8.4	48.1	4.82
31	Wheat	263	1.2	18	164	7.8	50.2	4.98
32	Barley	214	1.6	24	184	7.8	51.3	5.12
33	Spinach	198	5	63	112	8.2	52.5	5.69
34	Radish	225	2.3	18	95	8.1	47.9	5.38
35	Barley	212	1.6	89	116	8.4	45.7	5.50
36	Wheat	248	25	74	168	7.8	43.2	6.73
37	Barley	226	18	63	133	7.9	57.7	6.09
38	Barley	289	1.8	42	101.3	8.4	55.0	6.91
39	Alfalfa	251	2.2	33	116	8.3	56.1	7.29
40	Wheat	255	0.9	28	95.5	8.0	51.2	7.14
41	Barley	252	0.5	35	120.9	7.9	39.4	8.40
42	Barley	234	11	43	118	8.3	41.6	8.31
43	Wheat	196	8	75	131.6	8.5	52.3	8.06
44	Canola	268	0.7	25	124	8.2	55.2	3.85
45	Alfalfa	25	0.1	24	118.3	7.9	49.8	4.07

### Isolation of PGPR

For isolating PGPR, the collected soil samples were processed using a standard protocol (Rehan et al., 2023; Azadi and Shojaei, 2020). The rhizosphere soil was carefully separated from the roots using sterile brushes and passed through a 2 mm sieve. A soil suspension was prepared by suspending 10 g of soil in 100 mL of sterile saline solution (0.85% NaCl), followed by mixing in an orbital shaker at 28°C and 120 × g for 30 min to dislodge the rhizobacteria from soil particles. Serial tenfold dilutions were prepared from the homogenized suspensions (from 10^−^^2^ to 10^−4^), and 100 μL of each dilution was spread onto nutrient agar (NA) plates and incubated for 48 h at 25°C. Well-isolated colonies with distinct morphological characteristics were selected and subcultured on fresh NA plates to obtain pure cultures.

To assess the plant growth-promoting traits, the isolates were screened using specific media and biochemical assays:

- Phosphate solubilization was evaluated on Pikovskaya's agar (PKV) by observing clear halo zones around colonies.
- Siderophore production was assessed using Chrome Azurol S (CAS) agar, where a color change from blue to orange indicated positive results.
- Potassium solubilization was tested on Alexandrov agar, where solubilization zones confirmed activity.
- Ammonia production was determined using peptone water medium, followed by Nessler's reagent, which produced a yellow to brown color in positive isolates.
- Indole-3-Acetic Acid (IAA) production was assessed by culturing isolates in tryptophan-supplemented nutrient broth, followed by the addition of Salkowski reagent, where a pink color indicated IAA production.
- Zinc solubilization was tested on PKV medium supplemented with ZnO, where clear halo formation indicated solubilization ability.

After incubation under optimal conditions, isolates exhibiting positive results in these assays were selected for further phenotypic, biochemical, and molecular characterization. The well-isolated colonies with distinct morphology were further subjected to Gram staining, motility tests, catalase and oxidase activity tests, and 16S rRNA gene sequencing for precise taxonomic identification.

#### Microbiological identification of isolates

The selected isolates were characterized phenotypically by using the panel of conventional phenotypic and biochemical tests include, colony morphology, pigmentation, Gram staining, malachit green staining, hydrolysis of gelatin, motility test, catalase and oxidase production test (Ghaffari et al., 2024).

#### Molecular identification

Chromosomal DNA was extracted using boiling method described by Siavashifar et al. (2021) as follow: a few colonies of bacteria were added into 200 mL of TE buffer (Tris EDTA), boiled for 30 min and centrifuged at 10,000 rpm for 10 min. The supernatant was transferred to another sterile microtube and centrifuged at 13,000 rpm for 10 min. Precipitated DNA was re-suspended in 50 μl of Milli-Q water and stored at −20°C (Kumawat et al., 2023).

The PCR amplification and sequence analysis of almost complete of 16S rRNA gene was used for species identification of isolates as described by Fazelikia et al. (2023). Sequencing was performed by ABI 3100 genetic analyzer in Pishgam Biotech Company (Iran). The obtained sequences were aligned manually and compared and analyzed with all sequences of the closely related species retrieved from GenBank database using jPhydit program version 1.1.3 (Jeon et al., 2005).

#### Screening of PGPR

For the screening of plant growth-promoting bacteria, qualitative tests, including siderophore production, ammonia production, phosphate solubilization, potassium release, Indole-3-Acetic Acid (IAA) production and zinc solubilization were employed as described below.

#### Siderophore production capability test

Siderophore production capability of our isolates was conducted using the semi-quantitative CAS (Chrome Azurol S) method. The bacteria were initially cultured in Nutrient Broth (NB; HiMedia, India) for 48 h. Subsequently, 5 μL of a fresh bacterial suspension (10^8^ CFU mL^−1^) was inoculated into CAS medium and incubated at 28°C for 7 days. The presence of an orange halo around the colonies indicated siderophore production (Sultana et al., 2021).

#### Ammonia production capability test

Initially, the isolates were inoculated into tubes containing peptone water medium (comprising 10 g of peptone, 5 g of NaCl, and 5 g of yeast extract in one liter of water). The tubes were then incubated at 28°C for 5 days. After incubation, 0.5 mL of Nessler's reagent was added to each tube. The development of a brown to yellow color indicated a positive result for ammonia production by the bacteria (Agunbiade et al., 2024).

#### Phosphat solubilization capability test

Five microliters of a fresh bacterial suspension with a population density of 10^8^ CFU mL^−1^ were cultured on PKV (Pikovskaya) medium (Himedia, India), which contains 5 g/L of insoluble tricalcium phosphate. The inoculated plates were incubated at 28°C for 5 days. A clear zone around the colony was considered an indication of tricalcium phosphate solubilization (Agunbiade et al., 2024).

#### Potassium-releasing capability test

To determine the potassium-releasing ability, 5 μL of a fresh bacterial culture with a concentration of 10^8^ CFU mL^−1^ was added to Alexandrov agar medium (Himedia, India), which contains potassium aluminosilicate. The cultures were incubated at 28°C. The formation of a clear zone around the bacterial spot after 48 h indicated the bacteria's ability to solubilize and release potassium (Agunbiade et al., 2024).

#### Zinc soloubolization capability test

Five microliters of a fresh bacterial suspension with a concentration of 10^8^ CFU mL^−1^ was inoculated onto PKV medium containing 0.1% (1 g/L) ZnO and 5 g/L KH_2_PO_4_. The inoculated media were incubated at 28°C for 5 days. The formation of a clear zone around the colony was considered indicative of the solubilization of zinc compounds (Agunbiade et al., 2024).

#### IAA production capability test

Fifty microliters of the bacterial suspension were transferred to 25 mL of NB containing 100 mg/L of L-tryptophan. After 48 h, the bacterial suspension was centrifuged, and 1 mL of the supernatant was mixed with 2 mL of Salkowski's reagent. This mixture was then kept at room temperature for 25 min and OD was measured at a wavelength of 535 nm. The amount of IAA produced by each isolate was determined by comparing the absorbance values to a standard curve prepared using indole-3-acetic acid at concentrations of 0, 5, 10, 20, and 30 mg/L (Widowati et al., 2023).

### Analyzing the effect of PGPR on chamomile plant growth in salin condition

This experimental study was conducted in the greenhouse at Marvdasht Islamic Azad University, Iran. The greenhouse experiment was performed under controlled environmental conditions to ensure optimal plant growth and reproducibility of results. The temperature was maintained between 25 and 28°C, with relative humidity ranging from 65% to 75%. The plants were exposed to a 16-h light/8-h dark photoperiod, utilizing natural sunlight supplemented with artificial lighting when necessary to maintain uniform illumination. The greenhouse was well-ventilated to regulate airflow and prevent excessive humidity builduP. Soil moisture levels were carefully monitored, and irrigation was performed using distilled water to maintain 80% of the field capacity. These standardized conditions ensured a controlled environment for evaluating the effects of PGPR treatments on chamomile growth under saline conditions.

### Seed selection and germination testing

Chamomile seeds were obtained from the Shiraz Agriculture Research Institute, Shiraz, Iran. To ensure uniformity and consistency in plant growth, the seeds were tested for viability, uniformity, and germination rate before planting. Seeds were visually inspected, and only those with uniform size, shape, and color were selected, while damaged or irregular seeds were discarded.

Viability testing was performed using the tetrazolium chloride (TZ) test, in which randomly selected seeds were incubated in a 1% tetrazolium chloride solution at 25°C for 24 h. Only seeds with stained, viable embryos were selected for planting.

Germination testing was conducted by placing 100 randomly selected seeds on moist filter paper in Petri dishes under controlled conditions (25°C, 16-h light/8-h dark photoperiod). The germination percentage was recorded after 7 days, and only seeds with a germination rate of ≥85% were used in the experiment. These steps ensured that all planted seeds exhibited high viability and uniform germination, minimizing variability in plant growth responses.

### PGPR strain selection

A total of 181 bacterial isolates were initially screened for their plant growth-promoting (PGP) traits using phenotypic, biochemical, and molecular methods. The isolates were tested for phosphate solubilization, siderophore production, ammonia production, potassium solubilization, zinc solubilization, and IAA production. Out of these, 94 isolates exhibited at least one of the PGP traits, while only seven strains demonstrated all five major growth-promoting characteristics. These seven isolates were identified at the genus and species level using 16S rRNA sequencing. The selection was further refined based on their nutrient solubilization efficiency, ability to enhance plant biochemical parameters, and potential to support root development under saline conditions. These criteria ensured that the most effective PGPR strains were chosen for subsequent greenhouse experiments. Seven PGPR species isoted in our study include: *Bacillus cereus, P. fluorescens, P. syringae, Acinetobacter radioresistens, Pseudarthrobacter phenanthrenivorans, P. alcaliphila, Lysinibacillus macroides* were selected.

### Soil preparation

The soil used for this study was obtained from an agricultural area in southern Fars province of Iran with low salinity. The soil contained pH = 8.13, CaCO_3_ = 54%, EC = 2.12 dS.m^−1^, OC = 0.27%, N = 0.08%, P = 6.27 mg. kg^−1^, K = 185.60 mg. kg^−1^ and soil texture was sandy loam. To eliminate microbial contamination, the soil used in the experiment was sterilized using an autoclaving process at 121°C and 15 psi for 1 h. Since certain heat-resistant spores may survive a single autoclaving cycle, the sterilization procedure was repeated on two consecutive days to enhance microbial inactivation. To verify the effectiveness of sterilization, soil samples were tested for microbial contamination by plating on nutrient agar (NA) and potato dextrose agar (PDA). The plates were incubated at 28°C for 48 h, and the absence of bacterial and fungal growth confirmed the effectiveness of the sterilization process.

The greenhouse experiment was conducted in a factorial design and in a completely randomized design with three replications. The factor involved in study are: PGPR isolates: eight levels [p0 (control), p1 (*B. cereus*), p2 (*P. fluorescens*), p3 (*P. syringae*), p4 (*A. radioresistens*), p5 (*P. phenanthrenivorans*), p6 (*P. alcaliphila*), p7 (*L. macroides*)], Salinity: four levels (2, 4, 7, and 10 dS/m), Plant: Chamomile. To ensure unbiased results, the greenhouse experiment was conducted using a completely randomized design (CRD) with three replications for each treatment. The pots were randomly assigned to different positions within the greenhouse using a computerized randomization approach to eliminate any potential positional bias. Additionally, the placement of the pots was periodically rotated throughout the experiment to minimize microenvironmental variations, such as differences in light exposure, temperature distribution, and airflow patterns. Each treatment was labeled with coded identifiers to prevent observer bias during data collection.

### Pot experiment

The soil was dried in the air, sieved through a 6 mm mesh, then 5 kg of sterilized soil was added to each plastic pot (20 cm diameter, 25 cm height). Selected isolates were cultured in nutrient broth (NB) and incubated at 28°C for 24 h. The bacterial cultures were centrifuged at 6,000 rpm for 10 min at 4°C. From this, a bacterial suspension with a concentration of 108 CFU mL^−1^ was prepared by measuring optical density with a spectrophotometer. Then, seeds were surface sterilized with 5% sodium hypochlorite for 5 min, rinsed with distilled water, and dried. Afterward, seeds were inoculated with 500 μL of bacterial suspension (108 CFU mL^−1^) in NB for each treatment, while control seeds were immersed in 500 μL of sterile water. Inoculated seeds were planted after 5 h, with five seeds per pot, followed by thinning after germination to retain three uniform seedlings per pot. Finally, the pots were irrigated with distilled water to maintain the moisture level at 80% of the field capacity and were kept in the greenhouse for 8 weeks. Salinity treatments were induced using NaCl solutions at predefined EC levels (2, 4, 7, and 10 dS/m), gradually applied over 1 week through irrigation water to ensure uniform salinity levels across treatments.

### Analysis of vegetative and biochemical properties

After 8 weeks, plants were harvested. Shoot height and root length were measured with a ruler, and fresh and dry weights were determined after drying samples at 70°C for 48 h. Total chlorophyll, total carotenoids, proline, and protein content were measured based standard methods (Rasouli et al., 2023; Siddiqui et al., 2019).

### Analysis and nutrient uptake

Dry plant samples were ground and 0.5 g was ashed at 500°C for 6 h. Ash was dissolved in 2N HCl and diluted to 50 mL. Nutrient concentrations (sodium, potassium, calcium, magnesium, iron, zinc, manganese, and copper) were determined using flame photometry and atomic absorption spectroscopy, and phosphorus was measured colorimetrically using molybdo vanadate reagent. Nutrient uptake was calculated by multiplying dry weight by nutrient concentration. Post-harvest soil properties, including pH, electrical conductivity, organic carbon, sodium, calcium, magnesium, carbonates, bicarbonates, and sulfates, were measured using the same methods as pre-experiment analysis.

### Statistical analysis

In the analysis of the data, a one-way ANOVA was employed, followed by the comparison of means using Duncan's Multiple Range Test (DMRT). Prior to the execution of ANOVA, the Kolmogorov-Smirnov test was administered to confirm the normality of the data, while Levene's test was conducted to ensure the homogeneity of variances. The entirety of the data analysis was meticulously performed utilizing SAS software (version 9.4, SAS Institute Inc., Cary, NC).

## Results

After processing and culturing the 45 collected soil samples on specific media, a total of 181 bacterial isolates were identified through phenotypic, biochemical and molecular tests, include Gram staining, pigment production, catalase, oxidase, motility, IMViC test and PCR and seqences analaysis of 16SrRNA gene. Our results indicated that the isolates obtained in this study belonged to eight genera and 13 different species, including *B. cereus, P. fluorescens, P. syringae, A. radioresistens, P. phenanthrenivorans, P. alcaliphila, L. Macroides, B. Subtilis, N. Asteroides, N. Farcinica, T. denitrificanse, G. polyisoprenivorans*, and *B. sphaericus*.

**Figure 1:**
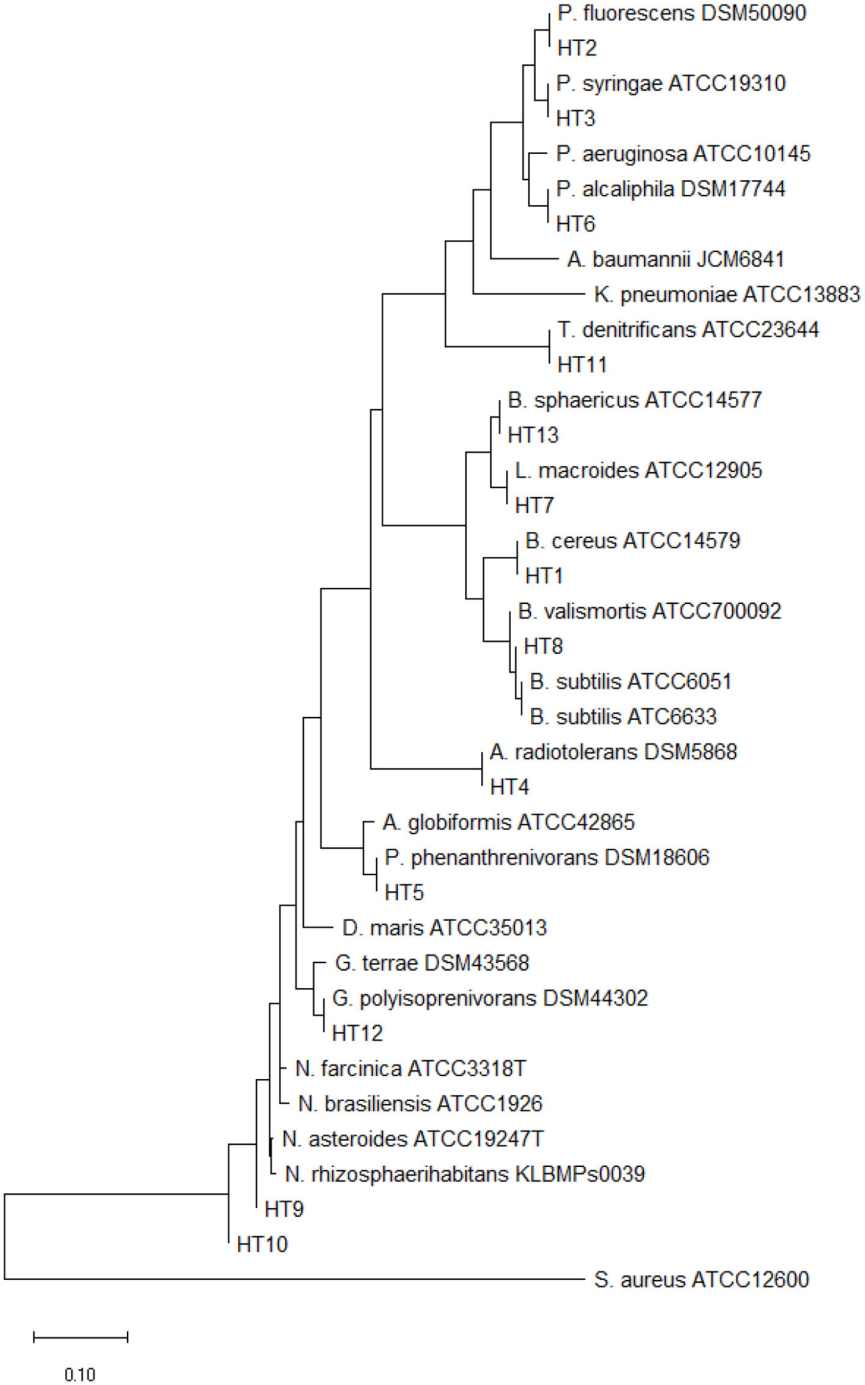
The phylogenetic tree was constructed by the neighbor-joining method using the MEGA 8 program with 1,000 bootstrap repetitions. *S. Aureus ATCC12600* is out-group.

The analysis of partial 16S rRNA gene sequences of our isolates showed that specific nucleotide signatures were present for each genus. For Gram positive bacteria, these signatures were observed at positions 70–98 (A-T), 307 (C), 293–304 (G-T), 614–626 (A-T), 631(G), 328 (T), 824e876 (T-A), 661–744 (G-C), 825–875 (A-T), 843 (C), and 1,122–1,151 (A-T), and for gram negative bacteria these signatures were observed at positions 70–98 (U-A), 843 (C), 1008–1021 (C-G), 139–224 (G-C), 1,189 (C), 1,308–1,329 (C-G), and 1244–129 (C-G) (Hassler et al., 2022). The relationship between our isolates and the standard Actinomycetes species was depicted by using a high bootstrap value phylogenetic tree of 16S rRNA gene by MEGA X software, using the neighbor-joining method with arithmetic mean of pairwise differences matrix (Figure 1).

### Nucleotide sequence accession numbers

The GenBank accession numbers for the 16S rRNA sequencing of isolated PGPR in this study are listed below. Isolate HT1 *Bacillus cereus* (OL946146), isolate HT2 *P. fluorescens* (OM019109.1), isolate HT3 P. syringae (OL979178.1), isolate HT4 *A. radiotolerans* (OM333625.1), isolate HT5 *P. phenanthrenivorans* (OL979177.1), isolate HT6 *P. alcaliphila* (OL979228.1), isolate HT7 *L. macroides* (OL979292.1), isolate HT8 *B. Subtilis* (PP956943), isolate HT9 *Nocardia Asteroides* (PP956956), isolate HT10 *N. Farcinica* (PP956957), isolate HT11 *Thiobacillus denitrifcanse*, isolate HT12 *Gordonia polyisoprenivorans* (PP957166), and isolate HT13 *B. sphaericus* (ON834526).

To assess the plant growth-promoting potential of the isolates, a series of tests were conducted, including siderophore production, ammonia production, phosphate solubilization, potassium release, IAA production, and zinc solubilization. The findings revealed that out of 181 isolates, 87 did not demonstrated any of the growth-promoting traits such as siderophore production, ammonia production, phosphate solubilization, potassium release, zinc solubilization and IAA production, while, 94 isolates showed between 1 and 5 growth-promoting properties. Conversely, 24 isolates demonstrated siderophore production, 40 showed ammonia production, 37 were capable of phosphate solubilization, 41 exhibited potassium release, 22 were able to solubilize zinc, and 22 produced IAA. Based on these comprehensive results, seven superior bacterial isolates, including *B. cereus, P. fluorescens, P. syringae, A. radioresistens, P. phenanthrenivorans, P. alcaliphila, L. macroides*, which possessed all five growth-promoting characteristics, were selected for further greenhouse experiments.

The results of the phenotypic, biochemical, molecular and plant growth promoting tests for each isolate are presented in Table 2.

**Table 2:** Phenotypic, biochemical, molecular, and plant growth promoting tests for each isolate.

**Species**	**No. isolates**	**Colony morphology-pigmentation**	**Gram staining**	**Resistance to lysosyme**	**Motility**	**Oxidase**	**Catalase**	**Gelatin hydrolysis**	**Siderophore production**	**Ammonia production**	**phosphate solubilization**	**Potassium solubilization**	**IAA production**	**16SrRNA base pair differences (%)**
*B. cereus*	16	Round-matte-creamy	–	–	+	+	+	+	+	+	+	+	+	2/800
*P. fluorescens*	21	Round-transparent-yellow	+	–	–	–	+	–	+	+	+	+	+	6/765
*P. syringae*	18	Round-matte-creamy	–	–	+	+	+	–	+	+	+	–	+	2/825
*A. radioresistens*	7	Round-matte-creamy	–	–	+	–	+	–	+	+	+	+	+	3/675
*P. phenanthrenivorans*	20	Spotted-transparent-yellow	–	–	+	–	+	–	+	+	+	+	+	2/814
*P. alcaliphila*	12	Spotted-matte-yellow	–	–	+	–	+	–	+	+	+	+	+	8/916
*L. macroides*	12	Round-matte-white	+	–	–	–	+	+	–	+	+	+	+	2/681
*B. subtilis*	14	Round-matte-creamy	+	–	+	+	+	+	–	+	+	–	+	3/758
*N. asteroides*	11	Irregular-yellow-powdery	+	+	–	+	+	–	–	+	+	–	–	2/743
*N. farcinica*	17	Round-matte-white	+	+	–	+	+	–	–	+	–	+	–	0/781
*T. denitrificanse*	12	Dotted-transparent-milky	–	–	–	–	–	–	–	–	+	+	–	3/816
*G. polyisoprenivorans*	5	Regular-matte-red	+	–	–	–	+	–	–	+	–	+	+	2/714
*B. sphaericus*	16	Round-matte-cream	+	–		+	+	+	–	+	–	+	+	0/821

### Analysis of vegetation and biochemical parameters of chamomile after PGPR traits

After isolating and identifying the bacteria, seven strains that showed the best results in plant growth promotion tests were selected and used in a pot experiment to study their effect on chamomile growth, following the methods section. After 8 weeks, the treated plants were harvested and analyzed for growth and biochemical parameters. The results indicated that all PGPR treatments significantly (*p* < 0.05) affected both vegetative and biochemical parameters of chamomile (*Matricaria chamomilla*; Table 3).

**Table 3:** Mean comparison of effect of the PGPR treatment on the vegetation and biochemical parameters of chamomile.

**Salinity**	**PGPR treatment**	**Root length (cm)**	**Shoot length (cm)**	**Root dry weight (g)**	**Shoot dry weight (g)**	**Total chlorophyll (mg/g)**	**Carotenoid (mg/g)**	**Proline (mg/g)**	**Essential oil (%)**
	Control	11.81 h	24.08 j	0.062 e	1.23 k	2.85 h	1.02 gh	0.22 f	0.61 gh
	P1 (*B. cereus*)	16.75 a	32.91 a	0.089 a	1.67 a	3.51 c	1.24 c	0.16 f	0.75 c
	P2 (*P. fluorescens*)	15.05 bc	29.89 c	0.079 b	1.51 c	3.87 a	1.38 a	0.15 f	0.83 a
	P3 (*P. syringae*)	14.89 c	26.72 e	0.079 b	1.36 e	3.14 e	1.13 d	0.16 f	0.68 de
2 (dS.m^−1^)	P4 (*A. radioresistens*)	13.53 d	25.01 g	0.071 c	1.26 g	2.95 g	1.06 f	0.19 f	0.64 f
	P5 (*P. phenanthrenivorans*)	11.90 gh	24.37 i	0.062 e	1.24 jk	2.85 hi	1.02 g	0.18 f	0.61 h
	P6 (*P. alcaliphila*)	13.23 e	24.67 h	0.070 c	1.25 h	2.91 g	1.04 fg	0.22 f	0.63 fg
	P7 (*L. macroides*)	13.63 d	27.49 d	0.072 c	1.40 d	3.23 d	1.15 d	0.16 f	0.70 d
	Control	8.28 k	21.57 m	0.044 g	1.10 o	2.55 l	0.92 jk	0.41 e	0.55 jk
	P1 (*B. cereus*)	15.21 b	30.51 b	0.081 b	1.55 b	3.07 f	1.10 e	0.32 e	0.67 e
	P2 (*P. fluorescens*)	11.85 gh	26.03 f	0.062 e	1.32 f	3.58 b	1.27 b	0.34 e	0.77 b
	P3 (*P. syringae*)	12.13 g	23.89 j	0.064 e	1.21 l	2.81 i	0.99 h	0.35 e	0.61 h
4 (dS.m^−1^)	P4 (*A. radioresistens*)	10.59 i	22.13 l	0.055 f	1.12 n	2.60 k	0.92 j	0.37 e	0.56 ij
	P5 (*P. phenanthrenivorans*)	10.10 j	22.91 k	0.054 f	1.16 m	2.70 j	0.96 i	0.38 e	0.58 i
	P6 (*P. alcaliphila*)	10.10 j	21.09 n	0.054 f	1.08 p	2.48 m	0.88 l	0.39 e	0.54 k
	P7 (*L. macroides*)	10.34 ij	24.37 i	0.054 f	1.24 ij	2.85 hi	1.02 gh	0.38 e	0.60 h
	Control	5.02 rs	19.23 s	0.026 mn	0.98 v	2.28 q	0.82 m	1.06 d	0.50 mn
	P1 (*B. cereus*)	12.62 f	24.56 hi	0.067 d	1.24 hi	2.42 n	0.88 l	0.96 d	0.52 lm
	P2 (*P. fluorescens*)	8.07 kl	20.77 o	0.043 g	1.06 r	2.88 h	1.04 fg	0.96 d	0.61 gh
	P3 (*P. syringae*)	10.22 j	19.81 q	0.054 f	1.01 t	2.32 p	0.85 m	0.97 d	0.50 mn
7 (dS.m^−1^)	P4 (*A. radioresistens*)	7.98 l	19.52 r	0.042 gh	0.99 u	2.31 pq	0.84 m	0.99 d	0.50 mn
	P5 (*P. phenanthrenivorans*)	6.95 n	21.17 m	0.037 i	1.07 q	2.49 m	0.89 kl	0.97 d	0.53 kl
	P6 (*P. alcaliphila*)	6.81 n	19.47 r	0.036 ij	1.00 u	2.28 q	0.83 m	1.04 d	0.49 n
	P7 (*L. macroides*)	7.86 l	19.95 q	0.041 h	1.01 t	2.34 op	0.84 m	1.03 d	0.50 mn
	Control	4.13 t	16.72 x	0.023 n	0.85 z	1.97 u	0.71 p	1.72 a	0.42 q
	P1 (*B. cereus*)	7.54 m	20.21 p	0.040 h	1.03 s	2.14 s	0.76 no	1.52 bc	0.46 op
	P2 (*P. fluorescens*)	6.11 o	18.27 u	0.032 kl	0.92 x	2.37 o	0.84 m	1.45 c	0.51 mn
	P3 (*P. syringae*)	5.30 qr	16.72 x	0.027 m	0.85 z	1.96 u	0.70 p	1.53 bc	0.42 q
10 (dS.m^−1^)	P4 (*A. radioresistens*)	6.23 o	17.57 w	0.034 j	0.89 z	2.07 t	0.75 o	1.61 b	0.45 p
	P5 (*P. phenanthrenivorans*)	5.67 p	18.61 t	0.030 l	0.94 w	2.18 r	0.79 n	1.55 bc	0.47 o
	P6 (*P. alcaliphila*)	5.58 pq	17.84 v	0.030 l	0.90 y	2.08 t	0.76 no	1.62 b	0.45 op
	P7 (*L. macroides*)	4.88 s	16.85 x	0.025 mn	0.85 z	1.97 u	0.70 p	1.49 c	0.43 q

Data are the mean ± SE values of three replicates. Means sharing the same letter (s) do not differ significantly (p ≤ 0.05), as determined by Duncan's multiple range test (n = 3).

The vegetation parameters of chamomile, including root and shoot length and dry weight of root and shoot, were significantly affected by all PGPR treatments and different salinity levels. The results were as follows: The P1 treatment at 2 dS.m^−1^ salinity showed the highest root length (16.75 cm) and shoot length (32.91 cm), while the control at 10 dS.m^−1^ had the lowest root (4.13 cm) and shoot length (16.72 cm). For dry root weight, P1 at 2 dS.m^−1^ had the highest (0.089 g), and the control at 10 dS.m^−1^ had the lowest (0.023 g). The dry shoot weight was highest in P1 at 2 dS.m^−1^ (1.67 g) and lowest in the control, P3, and P7 at 10 dS.m^−1^ (0.85 g). However, in all treatments, as the salinity levels increased from 2 to 10 dS.m^−1^, plant growth indices also showed a significant decrease (Table 3).

The biochemical parameters of chamomile, such as total chlorophyll, carotenoids, proline, and essential oil percentage, were notably affected by different PGPR treatments and salinity levels. Specifically, the P2 treatment at 2 dS.m^−1^ salinity demonstrated superior performance in terms of total chlorophyll content (3.87 mg/g fresh weight) and carotenoid production (1.38 mg/g fresh weight), whereas the control treatment at 10 dS.m^−1^ salinity displayed the highest proline production (1.72 μmol/g fresh weight). Moreover, the P2 treatment at 2 dS.m^−1^ salinity showed the highest essential oil production (0.83%), while, the lowest essential oil production seen in the P3, P7, and control treatments at 10 dS.m^−1^ salinity (0.42%, 0.42%, and 0.43%, respectively; Table 3). These results highlight the significant impact of PGPR treatments, on improving the biochemical composition of chamomile across varying salinity levels.

### Analysis of nutrients uptake of chamomile after PGPR traits

**Figure 2:**
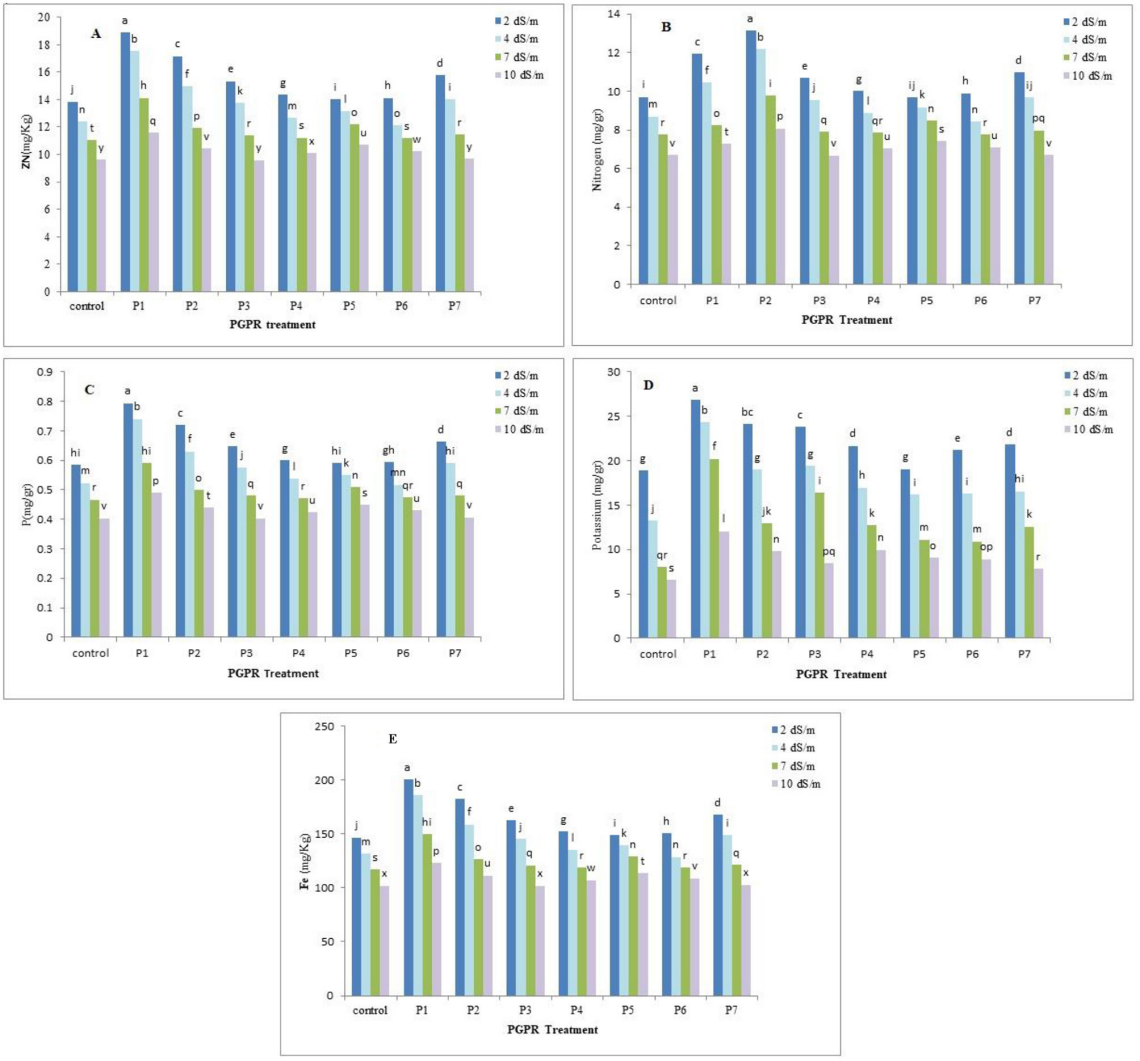
Effect of the PGPR treatment on the nutrients uptake by chamomile plant: **(A)** zinc uptake, **(B)** nitrogen uptake, **(C)** phosphorous uptake, **(D)** potassium uptake, **(E)** iron uptake. Different letter indicate analytical order in software for draw correct pattern.

The analysis of variance revealed that, the PGPR treatments significantly affected (*p* < 0.05) the uptake of nitrogen, phosphorus, potassium, iron, and zinc by chamomile plant, at the different salinity level. PGPR treatments notably increased nutrient concentrations compared to the control treatment. For nitrogen, the P2 treatment at 2 dS.m^−1^ showed the highest absorption (13.15 mg/g), while P3 at 10 dS.m^−1^ had the lowest (6.67 mg/g). Phosphorus absorption was highest with the P1 treatment at 2 dS.m^−1^ (0.79 mg/g), whereas P3, control, and P7 at 10 dS.m^−1^ had the lowest (0.40, 0.40, and 0.41 mg/g, respectively). Potassium absorption peaked with the P1 treatment at 2 dS.m^−1^ (26.80 mg/g) and was lowest in the control at 10 dS/m (6.61 mg/g). The P1 treatment at 2 dS.m^−1^ also exhibited the highest iron absorption (200.73 mg/kg), while the control and P3 at 10 dS.m^−1^ had the lowest (101.99 mg/kg). Zinc absorption was highest with the P1 treatment at 2 dS.m^−1^ (18.89 mg/kg), and lowest with P3, control, and P7 at 10 dS.m^−1^ (9.58, 9.62, and 9.67 mg/kg, respectively). PGPR inoculation under salinity stress significantly increased nutrient concentrations, particularly with the P1 and P2 treatments. However, as salinity increased from 2 to 10 dS/m, nutrient concentrations significantly decreased across all treatments. However, nutrients uptake decreased with increasing salinity stress in rhizobacterial treatments (Figure 2).

## Discussion

The growing global demand for food and the adverse impacts of climate change have intensified the need for sustainable agricultural practices that boost crop productivity while alleviating environmental stress (Molotoks et al., 2021). Among the various strategies, the use of PGPR has emerged as a promising solution. PGPR are beneficial bacteria that colonize plant roots and enhance growth through various mechanisms, including nutrient solubilization, phytohormone production, and pathogen suppression. These bacteria facilitate the availability and uptake of essential nutrients such as nitrogen, phosphorus, and potassium, thereby improving plant nutrition and growth, especially in nutrient-deficient soils (Bouremani et al., 2023). Additionally, PGPR play a crucial role in mitigating abiotic stresses like soil salinity, which significantly reduces agricultural productivity globally. By producing osmoprotectants and enzymes like ACC deaminase, PGPR enhance plant stress tolerance, ensuring sustained crop productivity in adverse conditions. Integrating PGPR into agricultural practices reduces dependency on chemical fertilizers and pesticides, aligning with sustainable agriculture principles and promoting soil health and biodiversity (Hasan et al., 2024). Thus, the significance of PGPR in sustainable agriculture is profound, offering a comprehensive approach to improving plant growth and resilience. Therefore, the aim of the present study was to isolate native PGPR from Irans farmlands and investigate the potential of various PGPR isolates to promote plant growth under different salinity levels, thereby identifying effective strains that could be utilized to enhance crop productivity in saline environments.

In this study we were isolated 181 bacterial strains from the rhizosphere of different plants grown under saline conditions and screened them for various growth-promoting traits. Of these isolates, 94 exhibited at least one growth-promoting property, while 87 showed none. Of these, seven isolates (*B. cereus, P. fluorescens, P. syringae, A. radioresistens, P. phenanthrenivorans, P. alcaliphilus*, and *L. macroides*) which exhibited all five growth-promoting characteristics were selected for chamomile growth promote experiment. Our results align with and build upon findings from other studies in the field of PGPR. The identification of diverse bacterial isolates, echoes the microbial richness observed in similar studies (Rehan et al., 2023; Slimani et al., 2023). However, what sets our study apart is the meticulous assessment of these isolates for their growth-promoting potential. We observed that seven superior bacterial isolates, possessing all five growth-promoting characteristics tested, showed promising results in terms of siderophore production, ammonia production, phosphate solubilization, potassium release, and IAA production. Our results, highlights the efficacy of specific PGPR strains in enhancing plant growth, which surpasses the findings of some previous studies that reported fewer isolates demonstrating such multifaceted growth-promoting traits. Therefore, our study contributes significantly to the knowledge base regarding PGPR applications in sustainable agriculture by identifying and validating potent bacterial strains that can positively influence crop productivity and nutrient uptake.

Our findings showed that different PGPR treatments significantly impacted (p <0.05) the biochemical and vegetation parameters and nutrient uptake by of chamomile under different salinity levels. Results was as follow:

Regarding vegetation parameters, P1 treatments at 2 dS.m^−1^ showed the highest root and shoot lengths. Additionally, P1 treatments at 2 dS.m^−1^ exhibited the highest dry weight of roots and shoots. As salinity levels increased from 2 to 10 dS.m^−1^, plant growth indices significantly decreased. Our study's results align with and contribute to the existing body of research on the effectiveness of PGPR treatments in promoting plant growth, particularly under varying salinity levels. Comparing our findings with similar studies by Kumawat et al. (2023) and Lumibao et al. (2022), we observed a consistent trend of PGPR treatments significantly impacting vegetative parameters such as root and shoot length, as well as dry weight of roots and shoots. However, what sets our study apart is the comprehensive evaluation of multiple PGPR strains, which allowed us to identify specific strains like P1 that exhibited superior performance in promoting chamomile growth, as evidenced by higher root and shoot lengths, and increased dry weights. These results suggest that our selected PGPR strains may offer better growth-promoting effects compared to some previous studies, demonstrating their potential for application in sustainable agriculture practices, especially in mitigating the adverse effects of salinity stress on plant growth.

In terms of biochemical parameters, P2 treatments at 2 dS.m^−1^ displayed the highest levels of chlorophyll and carotenoids. Moreover, the highest essential oil production was observed in P2 treatments. Conversely, the control treatment at 10 dS.m^−1^ exhibited the highest proline content. Thes results showed that, our findings, are consistent with similar research efforts by Ashry et al. (2022) and Slimani et al. (2023), highlighting the significant impact of PGPR treatments on improving the biochemical composition of plants under stress conditions. Specifically, our results demonstrate that the P2 treatment at 2 dS.m^−1^ salinity showed superior performance in enhancing total chlorophyll content and carotenoid production compared to other treatments, indicating its potential in promoting photosynthetic efficiency and plant health. Additionally, the control treatment at higher salinity levels exhibited increased proline production, which is a common response to stress in plants. Moreover, our study observed a notable increase in essential oil production with the P2 treatment, suggesting its potential in enhancing the medicinal properties of chamomile. Overall, our findings contribute to the growing body of evidence supporting the positive effects of PGPR treatments on plant biochemistry, paving the way for their practical application in sustainable agriculture and herbal medicine.

Regarding nutrient uptake analysis, PGPR treatments significantly increased the absorption of nitrogen, phosphorus, potassium, iron, and zinc. P1 treatments at 2 dS.m^−1^ showed the highest absorption rates for most nutrients. However, nutrient concentrations decreased with increasing salinity stress across all treatments. These finding are in line with research by Ateş et al. (2022) and Dashti et al. (2021), demonstrating the significant role of PGPR in improving nutrient concentrations in plants, especially under salinity stress. The P1 and P2 treatments, in particular, showed notable increases in nitrogen, phosphorus, potassium, iron, and zinc absorption compared to the control treatment. This suggests the efficacy of these PGPR strains in enhancing nutrient availability and uptake by chamomile plants. However, it's essential to note that as salinity levels increased, there was a general trend of decreased nutrient concentrations across all treatments, indicating the challenging nature of saline environments on nutrient absorption. Despite this, our study's results highlight the potential benefits of PGPR inoculation in mitigating nutrient deficiencies and enhancing plant nutrition, contributing positively to sustainable agricultural practices.

## Conclusion

In conclusion, the results of our study demonstrate that soil salinity has significant negative effects on the growth and nutrient uptake indices of plants. However, inoculation with growth-promoting bacteria (PGPR) isolated from the rhizosphere of native plants in calcareous soils enhances plant access to water and soil nutrients, thereby improving the growth indices of chamomile plants. Furthermore, these bacterial isolates increase the concentrations of osmotic regulatory compounds, polyamines, and antioxidant enzyme activity in both the aerial parts and roots of plants, especially under saline conditions. This is achieved by enhancing nutrient concentrations, particularly nitrogen, phosphorus, potassium, iron, and zinc. The PGPR isolates also play a crucial role in reducing the levels of active oxygen species and free radicals, which ultimately strengthens plant resistance to salinity stress. Therefore, it can be concluded that these strains not only enhance the availability of macro- and micronutrients in the soil but also improve growth indices and biochemical parameters of the plants. Based on the findings of this research, the application of PGPR strains has proven to be highly effective in enhancing the studied parameters and improving the resistance of chamomile and alfalfa plants to salinity stress. These results highlight that the use of native soil rhizobia with strong growth-promoting characteristics, as biological fertilizers, can reduce the reliance on chemical fertilizers, offering an environmentally friendly alternative for enhancing plant growth and stress resistance.

## Data Availability

The datasets presented in this study can be found in online repositories. The names of the repository/repositories and accession number(s) can be found below: https://www.ncbi.nlm.nih.gov/genbank/, OL946146; https://www.ncbi.nlm.nih.gov/genbank/, OM019109; https://www.ncbi.nlm.nih.gov/genbank/, OL979178; https://www.ncbi.nlm.nih.gov/genbank/, OM333625; https://www.ncbi.nlm.nih.gov/genbank/, OL979177; https://www.ncbi.nlm.nih.gov/genbank/, OL979228; https://www.ncbi.nlm.nih.gov/genbank/, OL979292; https://www.ncbi.nlm.nih.gov/genbank/, PP956943; https://www.ncbi.nlm.nih.gov/genbank/, PP956956.
